# Outcomes Following eHealth Weight Management Interventions in Adults With Overweight and Obesity From Low Socioeconomic Groups: Protocol for a Systematic Review

**DOI:** 10.2196/34546

**Published:** 2022-01-20

**Authors:** Richard Myers-Ingram, Jade Sampford, Rhian Milton-Cole, Gareth David Jones

**Affiliations:** 1 Department of Physiotherapy Guy's and St Thomas' NHS Foundation Trust London United Kingdom; 2 Department of Population and Health Sciences King’s College London London United Kingdom

**Keywords:** obesity, eHealth, technology, weight management, weight loss, low socioeconomic status, socioeconomic, systematic review, weight, obese

## Abstract

**Background:**

Obesity is a complex health condition with multiple associated comorbidities and increased economic costs. People from low socioeconomic status (SES) backgrounds are more likely to be overweight and obese and are less successful in traditional weight management programs. It is possible that eHealth interventions may be more successful in reaching people from low SES groups than traditional face-to-face models, by overcoming certain barriers associated with traditional interventions. It is not yet known, however, if eHealth weight management interventions are effective in people living with overweight and obesity from a low SES background.

**Objective:**

The primary aim of this study is to evaluate the efficacy of eHealth weight management interventions for people with overweight and obesity from low SES groups.

**Methods:**

A systematic review on relevant electronic databases (MEDLINE, Embase, Emcare, and CINAHL) will be undertaken to identify eligible studies published in English up until May 2021. Using the Preferred Reporting Items for Systematic Reviews and Meta-Analysis (PRISMA) statement to guide the systematic review, two reviewers will independently screen, select, and extract data and complete a risk of bias assessment of search results according to predefined criteria. Studies that have investigated an eHealth weight management intervention within a low SES population will be included. Primary outcomes include weight, BMI, and percentage weight change compared at baseline and at least one other time point. Secondary outcomes may include a range of anthropometric and physical fitness and activity measures. If sufficient studies are homogeneous, then we will pool results of individual outcomes using meta-analysis.

**Results:**

Searches have been completed, resulting in 2256 studies identified. Once duplicates were removed, 1545 studies remained for title and abstract review.

**Conclusions:**

The use of eHealth in weight management programs has increased significantly in recent years and will continue to do so; however, it is uncertain if eHealth weight management programs are effective in a low SES population. The results of this systematic review will therefore provide a summary of the evidence for interventions using eHealth for people living with overweight and obesity and from a low SES background.

**Trial Registration:**

PROSPERO International Prospective Register of Systematic Reviews CRD42021243973; https://tinyurl.com/2p8fxtnw

**International Registered Report Identifier (IRRID):**

DERR1-10.2196/34546

## Introduction

### Background

Overweight and obesity are associated with increased risk of developing common diseases that can cause premature death. These include type 2 diabetes, cardiovascular disease, liver disease, and some cancers and respiratory diseases [1]. There is also a bidirectional association between obesity and depression [2]. Overweight and obesity is therefore a complex condition conferring significant health, social care, and financial burden, which requires carefully designed countermeasures and interventions throughout the life cycle [3]. A person is currently considered overweight if their BMI (the ratio of mass in kg to squared height in meters) is ≥25kg/m^2^, and obese if their BMI is ≥30 kg/m^2^ [4]. Overweight and obesity is a growing health problem. Global obesity rates have more than doubled since 1980, with the mean (95% CI) BMI in the United Kingdom rising from 24.7 (24.4-25.0) kg/m^2^ in 1986 to 27.1 (26.8-27.5) kg/m^2^ in 2016 [5]. Tackling overweight and obesity is therefore an urgent public health emergency for policy makers, clinicians, and researchers, as well as the individual themselves [6,7].

Independent studies globally have confirmed that low socioeconomic status (SES) is associated with increased rates of overweight and obesity (eg, in China [8], the United States and France [9], and the United Kingdom [10]). Several parameters need to be assessed to measure SES including an individual’s income, educational level, and occupation [11]. Other measures exist such as the Indices of Deprivation, used in England, which also includes exposure to crime, health, housing, and living environment domains [12]. These measures have been used to provide compelling evidence that deprivation is associated with worse health behaviors and outcomes; those who live in more deprived areas are more likely to engage in unhealthy behaviors (smoking, increased alcohol consumption) and less likely to engage in healthy behaviors (physical activity, healthy diet) compared with those in less deprived areas [13]. Thus, SES should be considered a confounding factor in studies determining the efficacy of weight management interventions if the intervention adopts behavior change approaches [6].

It is not surprising therefore that traditional behavior change interventions that target unhealthy behaviors in low SES groups have reported modest improvements in weight and physical fitness compared to people from higher SES. For example, meta-analyses estimated modest standardized mean differences (95% CI) between low-income groups and controls of 0.22 (0.14-0.29) following diet interventions, 0.21 (0.06-0.36) following physical activity interventions, and a relative risk of smoking following abstinence interventions of 1.59 (1.34-1.89) [14]. In a qualitative study of people delivering, receiving, and following an eating lifestyle change intervention in a low SES community, incorporation of diverse language/literacy, cultural origin, and the availability/cost of healthy foods and physical activity options were important factors that could lead to more equitable success in weight loss [15]. This study acknowledged that not adapting interventions that are less efficacious for people with low SES would increase existing inequalities across SES groups. However, it also acknowledged that a dearth of literature is available with which to plan what to adapt (and how), and its results certainly help to address this by tailoring the service to multiple cultures and lobbying for fair local amenity access. What this welcome study did not include as a factor was any personal choice in the medium of weight loss interventions.

eHealth is defined as interventions delivered using computers, mobile phones, or similar media devices via internet websites/web applications; mobile or social network apps; email; or SMS text messaging [16]. It is possible that technological advances mean eHealth interventions can be offered as an alternative approach for low SES patients. Traditional weight management interventions typically require frequent face-to-face sessions. However, individuals from low SES have expressed barriers to physically attending health care appointments, which include stress arising from taking time off work, and excessive travel and childcare costs within limited personal budgets. It is therefore possible that eHealth options may be preferred by low SES individuals [17,18]. Prioritizing the use of technology may also help reach diverse groups that are often underrepresented in research and real-world interventions [19].

Ambitions for eHealth have been transparent in the UK National Health Service (NHS) over the last decade. NHS England’s Five Year Forward View alluded to the health care opportunities afforded by the “information revolution” and “electronic glue” [20]. More recently, one of the NHS Long Term Plan aims was to increase the percentage of “digital access” options for services available for patients’ care, with 100% of patients being offered a “digital-first” primary care consultation by 2023/24 [21]. There is also the acceleration of eHealth uptake in response to the COVID-19 pandemic, culminating in recent guidance from NHS England that 25% of all outpatient health consultations in secondary care should be offered remotely via telephone or video [22]. Given these ambitions and the eHealth evolution, there is no doubt the direction of travel for weight management interventions incorporates eHealth options. Although a large meta-analysis demonstrated that eHealth weight management interventions achieve statistically significant weight loss compared to controls [19], the analyses did not account for SES. Thus, it remains unclear whether eHealth options could lead to more equity in weight management intervention outcomes independent of SES.

### Objectives

We propose to undertake a systematic review of the literature and determine what eHealth weight management interventions are offered and whether they are effective in facilitating weight loss and physical fitness and activity gains in people with low SES. The primary aim of this study is to first determine what eHealth weight management interventions exist in promoting weight loss and improving physical activity and fitness for people living with overweight and obesity from low SES groups, and second evaluate their efficacy. The proposed systematic review aims to answer the following questions:

1. Are eHealth interventions effective in facilitating weight loss in people living with overweight or obesity from a low SES background?
2. Are eHealth interventions effective in facilitating improved physical fitness in people living with overweight or obesity from a low SES background?

## Methods

### Overview

This systematic review will be conducted in accordance with the PRISMA (Preferred Reporting Items for Systematic Reviews and Meta-Analysis) statement [23]. The protocol has been reported according to the PRISMA-P (Preferred Reporting Items for Systematic Reviews and Meta-Analysis Protocols) checklist [24] (Multimedia Appendix 1).

### Eligibility Criteria

Eligibility criteria were structured using a PICOS (Population, Intervention, Comparison, Outcomes, and Study design) framework [25] (Table 1).

**Table 1:** Study eligibility criteria using the PICOS (Population, Intervention, Comparison, Outcomes, and Study design) criteria.

Criteria	Inclusion	Exclusion
Population	Adults ≥18 years old with BMI >25 kg/m^2^ Low socioeconomic status	Pregnancy or postpartum (within 3 months) Any socioeconomic status other than low socioeconomic status
Intervention	Weight management intervention delivered using eHealth technology	Bariatric surgery Medication-only interventions
Comparator	N/A^a^	N/A
Outcome	Weight (kg), BMI (kg/m^2^), and/or percentage weight change A range of anthropometric and physical fitness measures	N/A
Study design	Experimental studies Observational studies Case studies/series	Reviews Secondary analysis

^a^N/A: not applicable.

### Population

Adults aged ≥18 years who are living with overweight or obese (BMI ≥25 kg/m^2^) at baseline and are from a low SES background will be included. Low SES will include either low educational level, low income, or low occupational status, or any combination of these [11] (Table 2). The term “low” will refer to less well paid occupational status, fewer years of academic study, and lower income or a similar social disadvantage [26]. Individual studies will need to have defined SES based on stated criteria to be included. Studies including adults and children may be considered if data reported for adults are recorded separately. All participants with comorbidities will be included due to known associations of overweight and obesity with other health conditions [27].

**Table 2:** Outline of domains that relate to socioeconomic status.

Domain	Explanation
Income	The earnings an individual or family receive from employment, generally compared against the nation’s average earnings [28]
Education	An indicator for knowledge and involves the level of educational attainment, generally measured as the highest level of schooling achieved, such as primary, secondary, and tertiary education [26]
Occupational status	Involves the power, income, and educational requirements associated with the job role itself and the physical or hazardous demands related to that job [29]

### Intervention Types

Studies will be included if their weight management intervention aims include weight loss, weight loss maintenance, and physical fitness, and/or physical activity increase. Intervention mechanisms will be included if they include those recommended by the National Institute for Health and Care Excellence (NICE) guidelines [30] for weight management programs and include behavior change, diet, and nutrition education, meal replacement interventions, physical activity advice, and activity and exercise. Studies that involve one or more domain as outlined by NICE [30] will be considered for inclusion as previous literature has identified large variations in published eHealth interventions [16,19]. Interventions will be eligible if they include a single or range of eHealth technology to deliver content, which may be delivered via the web, mobile apps, mobile phones, computers, or other related devices that require participants to be engaged. We will exclude studies involving bariatric surgery or obesity medication only interventions.

### Comparisons

No limitation will be imposed on the control group. Studies with or without a control group will be considered eligible.

### Outcomes

The primary outcomes of interest are weight (kg) and BMI (kg/m^2^) either in absolute or proportional terms. Secondary outcomes will include any anthropometric or fitness measures including (but not limited to) body composition, percentage change of lean muscle mass, VO_2max_ (maximum oxygen consumption), estimated VO_2max_, predicted VO_2peak_ (volume of oxygen uptake during peak exercise), aerobic capacity, and physical activity levels. We have included a large range of outcomes to counter the expected large variation among studies. A combination of anthropometric measures and cardiorespiratory fitness measures have been considered to ensure a breadth of results are included. All outcomes will be included for data extraction if secondary measurement has been made in addition to baseline to evaluate the effect of the intervention.

### Study Design

We will include experimental and observational cohort studies designed to describe and/or investigate the efficacy of eHealth interventions. Experimental trials will include randomized control trials (RCTs), controlled clinical trials, or cluster trials. Observational studies will include prospective and retrospective comparative cohort studies, and cross-sectional, case-control, or nested case-control studies. We will exclude review articles, secondary analysis, and case study articles.

### Timing

There will be no restrictions on the length of follow-up of outcomes.

### Setting

There will be no restrictions by type of setting as interventions will be by remote access.

### Language

Only studies written in the English language will be included.

### Search Strategy

Literature search strategies will be developed using medical subject headings (MeSH) and text words related to the eligibility criteria outlined (Multimedia Appendix 2). We will search MEDLINE, Embase, Emcare, and CINAHL electronic databases. Both subject header and free-text searches will be completed, using Boolean search techniques, based on our PICOS framework (Table 1). Weight management and eHealth search terms were based on a previously published systematic review [16]. The grey literature will be searched using OpenGrey [31], and completed master’s and doctoral theses will be searched using E-Theses Online Service (EThOS) [32]. All databases will be searched from their respective inception dates.

### Data Collection and Analysis

#### Study Selection

Two authors (JS and RMI) will complete the database searches using the search terms in Multimedia Appendix 3. Results from the database searches will be transferred to proprietary reference manager software (Endnote X8.0.1, Clarivate) and duplicates will be removed. Proprietary systematic review software (Rayyan Systems Inc) will be used by the same two authors to independently screen titles, abstracts, and full-text articles according to the eligibility criteria. Reasons for exclusion will be explained and discrepancies will be resolved by a third experienced reviewer (GDJ) if consensus cannot be reached by the two authors.

#### Data Extraction

Literature search results will be collated in an adapted data extraction form based on The Cochrane Data Extraction Form for RCTs and non-RCTs [33]. Extracted data categories are outlined in Textbox 1. Two authors (JS and RMI) will independently extract data, with any discrepancies settled by a third experienced reviewer (GDJ) if consensus cannot be reached by the two authors.

**Data extraction form checklist.**

**Source**

- Reviewer name
- Review date
- Study title and authors
- Journal name
- Publication date

**Eligibility**

- Confirm eligibility for review

**Methods: participants**

- Setting (including country)

**Methods: design**

- Study design and duration

**Methods: interventions**

- Description of intervention (eg, diet/nutrition advice, physical activity, behavior change techniques)
- Length of intervention/follow-up
- Delivery details (type of eHealth, eg, internet-based, social media, mobile phone/app, online platforms, emails, texts)

**Methods: outcomes**

- Name and definition (eg, weight [kg], BMI [kg/m^2^], and/or percentage weight change, a range of anthropometric and physical fitness measures)
- Time point measure
- Attrition rate

**Results**

- Number of participants randomized/allocated per group/analyzed
- Baseline characteristics (age, ethnicity, sex, weight, BMI, socioeconomic status)
- Summary data for each group at each time point
- Any adverse events

#### Quality

Two authors (RMI and JS) will independently assess the risk of bias using the Joanna Briggs Institute (JBI) checklist [34,35]. The article study design will influence what element of the JBI checklist we use, but it is anticipated that most included studies will be of cross-sectional design, therefore it is likely that the JBI Checklist for Analytical Cross Sectional Studies will be used. The JBI checklist uses a 3-point nominal rating scale, where a score of 0 is assigned for low risk of bias, 1 for unclear, and 2 for high risk of bias for each of the domains on the checklist. Overall, a high risk of bias will be concluded if a study returned a final rating of >50% of the total possible score. For example, the JBI Checklist for Analytical Cross Sectional Studies has 8 domains; therefore, a high risk of bias will equal a score of 8 or more. See Multimedia Appendix 4 for each JBI critical appraisal tool and related domains and descriptions. Disagreements between reviewers will be resolved by a third author (GDJ) if consensus cannot be reached.

### Data Analysis

We will conduct a narrative synthesis on all available data, examining findings between and within studies following national guidelines [25]. The narrative synthesis will include an account of interventions (eg, eHealth), participants’ characteristics, and outcomes.

If an adequate number of homogeneous studies in terms of participants, intervention, and outcomes are returned, the individual outcomes will be pooled quantitatively using a fixed- and random-effects meta-analysis.

### Amendments

In the event that the protocol needs amending, we will provide dates of each amendment, describe the changes, and give rationale in the section.

## Results

This study aims to determine the effectiveness of weight management programs delivered using eHealth for people living with overweight and obesity and from a low SES. Searches were completed on May 5, 2021, in the 4 selected databases and 2256 studies have been identified. Once duplicates were removed, 1545 studies remained for title and abstract review.

## Discussion

The prevalence of people living with overweight and obesity has increased over time. Although eHealth has been an effective tool in weight management programs, it is not yet clear if eHealth weight management interventions are effective for people with low SES. The results of this systematic review will therefore provide a summary of the evidence for interventions using eHealth for people living with overweight and obesity and from a low SES. If the results are not definitive, the systematic review will identify where further research is required.

## Appendix

Multimedia Appendix 1 PRISMA-P (Preferred Reporting Items for Systematic Reviews and Meta-Analysis Protocols) checklist.

Multimedia Appendix 2 Search terms using PICOS (Population, Intervention, Comparison, Outcomes, and Study design) criteria.

Multimedia Appendix 3 Database search overview.

Multimedia Appendix 4 Risk of bias assessment tools.

## Abbreviations

EThOS: E-Theses Online Service
JBI: Joanna Briggs Institute
NHS: National Health Service
NICE: National Institute for Health and Care Excellence
PICOS: Population, Intervention, Comparison, Outcomes, and Study design
PRISMA: Preferred Reporting Items for Systematic Reviews and Meta-Analysis
PRISMA-P: Preferred Reporting Items for Systematic Reviews and Meta-Analysis Protocols
RCT: randomized controlled trial
SES: socioeconomic status
VO_2max_: maximum oxygen consumption
VO_2peak_: volume of oxygen uptake during peak exercise

## References

[1] Guh DP, Zhang W, Bansback N, Amarsi Z, Birmingham CL, Anis AH (2009). The incidence of co-morbidities related to obesity and overweight: a systematic review and meta-analysis. BMC Public Health.

[2] Luppino FS, de Wit LM, Bouvy PF, Stijnen T, Cuijpers P, Penninx BWJH, Zitman FG (2010). Overweight, obesity, and depression: a systematic review and meta-analysis of longitudinal studies. Arch Gen Psychiatry.

[3] Wang YC, McPherson K, Marsh T, Gortmaker SL, Brown M (2011). Health and economic burden of the projected obesity trends in the USA and the UK. Lancet.

[4] National Institute of Health and Care Excellence (2014). Obesity: identification, assessment and management (CG189).

[5] World Health Organization (2021). Prevalence of overweight among adults, BMI >= 25 (crude estimate) (%). The Global Health Observatory.

[6] Loring B, Robertson A (2014). Obesity and inequities: Guidance for addressing inequities in overweight and obesity.

[7] Theis DRZ, White M (2021). Is obesity policy in England fit for purpose? Analysis of government strategies and policies, 1992-2020. Milbank Q.

[8] Zhang H, Xu H, Song F, Xu W, Pallard-Borg S, Qi X (2017). Relation of socioeconomic status to overweight and obesity: a large population-based study of Chinese adults. Ann Hum Biol.

[9] Drewnowski A, Moudon AV, Jiao J, Aggarwal A, Charreire H, Chaix B (2014). Food environment and socioeconomic status influence obesity rates in Seattle and in Paris. Int J Obes (Lond).

[10] Bann D, Johnson W, Li L, Kuh D, Hardy R (2017). Socioeconomic inequalities in body mass index across adulthood: coordinated analyses of individual participant data from three British birth cohort studies initiated in 1946, 1958 and 1970. PLoS Med.

[11] Baker E, Cockerham WC, Dingwall R, Quah S (2014). Socioeconomic Status, Definition. The Wiley Blackwell Encyclopedia of Health, Illness, Behavior, and Society.

[12] (2019). English indices of deprivation.

[13] Giskes K, Avendano M, Brug J, Kunst AE (2010). A systematic review of studies on socioeconomic inequalities in dietary intakes associated with weight gain and overweight/obesity conducted among European adults. Obes Rev.

[14] Bull ER, Dombrowski SU, McCleary N, Johnston M (2014). Are interventions for low-income groups effective in changing healthy eating, physical activity and smoking behaviours? A systematic review and meta-analysis. BMJ Open.

[15] Coupe N, Cotterill S, Peters S (2018). Tailoring lifestyle interventions to low socio-economic populations: a qualitative study. BMC Public Health.

[16] Bennett GG, Steinberg DM, Stoute C, Lanpher M, Lane I, Askew S, Foley PB, Baskin ML (2014). Electronic health (eHealth) interventions for weight management among racial/ethnic minority adults: a systematic review. Obes Rev.

[17] Rosenbaum DL, Piers AD, Schumacher LM, Kase CA, Butryn ML (2017). Racial and ethnic minority enrollment in randomized clinical trials of behavioural weight loss utilizing technology: a systematic review. Obes Rev.

[18] Thomas JG, Bond DS (2014). Review of innovations in digital health technology to promote weight control. Curr Diab Rep.

[19] Hutchesson MJ, Rollo ME, Krukowski R, Ells L, Harvey J, Morgan PJ, Callister R, Plotnikoff R, Collins CE (2015). eHealth interventions for the prevention and treatment of overweight and obesity in adults: a systematic review with meta-analysis. Obes Rev.

[20] National Health Service England (2014). Five Year Forward View.

[21] National Health Service (2019). The NHS Long Term Plan.

[22] National Health Service NHS 2021/22 Priorities and Operational Planning Guidance.

[23] Page MJ, McKenzie JE, Bossuyt PM, Boutron I, Hoffmann TC, Mulrow CD, Shamseer L, Tetzlaff JM, Akl EA, Brennan SE, Chou R, Glanville J, Grimshaw JM, Hróbjartsson A, Lalu MM, Li T, Loder EW, Mayo-Wilson E, McDonald S, McGuinness LA, Stewart LA, Thomas J, Tricco AC, Welch VA, Whiting P, Moher D (2021). The PRISMA 2020 statement: an updated guideline for reporting systematic reviews. Syst Rev.

[24] Moher D, Shamseer L, Clarke M, Ghersi D, Liberati A, Petticrew M, Shekelle P, Stewart LA, PRISMA-P Group (2015). Preferred reporting items for systematic review and meta-analysis protocols (PRISMA-P) 2015 statement. Syst Rev.

[25] Centre for Reviews and Dissemination (2009). Systematic Reviews: CRD's guidance for undertaking reviews in health care.

[26] Robertson A, Lobstein T, Knai C (2007). Obesity and socio-economic groups in Europe: Evidence review and implications for action.

[27] Abdelaal M, le Roux CW, Docherty NG (2017). Morbidity and mortality associated with obesity. Ann Transl Med.

[28] APA Task Force on Socioeconomic Status (2007). Report of the APA Task Force on Socioeconomic Status.

[29] Geyer S, Peter R (2000). Income, occupational position, qualification and health inequalities-competing risks? (comparing indicators of social status). J Epidemiol Community Health.

[30] National Institute for Health and Care Excellence (2014). Weight management: lifestyle services for overweight or obese adults.

[31] Dumouchel B, Dercourt J (2009). Assessing the quality and impact of research: Practices and initiatives in scholarly communication. ISU.

[32] Paez A (2017). Gray literature: An important resource in systematic reviews. J Evid Based Med.

[33] Li T, Higgins JPT, Deeks JJ, Higgins JPT, Thomas J, Chandler J, Cumpston M, Li T, Page MJ, Welch VA (2019). Collecting Data. Cochrane Handbook for Systematic Reviews of Interventions, Second Edition.

[34] Moola S, Munn Z, Tufanaru C, Aromataris E, Sears K, Sfetcu R, Aromataris E, Munn Z (2020). Chapter 7: Systematic reviews of etiology and risk. JBI Manual for Evidence Synthesis.

[35] Tufanaru C, Munn Z, Aromataris E, Campbell J, Hopp L, Aromataris E, Munn Z (2020). Chapter 3: Systematic reviews of effectiveness. JBI Manual for Evidence Synthesis.

