# Supplementary material for: Outcomes Following eHealth Weight Management Interventions in Adults With Overweight and Obesity From Low Socioeconomic Groups: Protocol for a Systematic Review
Source: JMIR Res Protoc. 2022 Jan 20;11(1):e34546. doi: 10.2196/34546 (PMC8814919; doi:10.2196/34546)
Supplement: Multimedia Appendix 2 [file resprot_v11i1e34546_app2.docx]

## Multimedia Appendix- Search terms using PICOS criteria

**Table 1.** Search terms to be used within the systematic review using PICOS criteria

| Population | Intervention | Comparator | Outcome | Filters |
| --- | --- | --- | --- | --- |
| Obesity (MESH)  Obese (MESH)  Overweight  Adult  ‘Social disparit$’ (MESH)  ‘Social inequalit$’ (MESH)  ‘Social inequity’  ‘Economic disparity’  ‘Economic inequality’  ‘Economic inequity’  ‘Socio-economic disparity’  ‘Socioeconomic disparity’  ‘Socioeconomic inequality’  ‘Socio-economic inequality’  ‘Socioeconomic inequity’  ‘Socio-economic inequity’  ‘Low income’  ‘Low education’  Employment (MESH) | e-health (MESH)  ‘electronic health’  ‘telemedicine’  ‘telehealth’  ‘mhealth’  ‘m-health’  ‘mobile health’  ‘interactive media’  ‘telephone’  ‘telephone-based’  ‘phone-based’  ‘internet’  ‘internet-based’  web  ‘web-based’  ‘website’  ‘website-based’  ‘email’  ‘electronic mail’  ‘e-mail-based’  ‘computers’  ‘computer’  ‘computer-based’  ‘wireless’  ‘mobile phone’  ‘cell phone’  ‘cellular phone’  ‘smartphone’  ‘mobile device’  ‘personal digital assistant’  ‘pda’  ‘interactive voice response’  ‘ivr’  ‘text message’  ‘text messaging’  ‘SMS’  ‘bluetooth’  ‘chat’  ‘chat room’  ‘instant message’  ‘IM’  twitter  tweet  ‘blog’  ‘social network’  tailored  automated  ‘individualized  programme’  “individualised  programme’  remote  ‘self-monitoring’  ‘feedback’ ‘prompt’  ‘reminder’  AND  “weight loss program*”  Diet (MESH)  Diet  Nutrition (MESH)  Nutrition  “Physical activity”  Exercise (MESH)  Exercise  “weight management program*”  “weight management intervention”  “weight loss intervention”  “behaviour change”  “behaviour change techniques”  “Low calorie diet”  “very low calorie ketogenic diet” | N/A | ‘Body mass index’ (MESH)  ‘Body mass index’  ‘BMI’  ‘weight loss’ (MESH)  ‘weight loss’  ‘weight reduction’(MESH)  ‘weight reduction program*’  ‘weight loss maintenance’  ‘weight gain prevention’  ‘obesity trials’  ‘obesity reduction’  ‘obesity prevention’  ‘cardiorespiratory fitness’  ‘cardiopulmonary fitness’  ‘cardiovascular fitness’  ‘VO2max’  ‘estimated VO2max’  ‘predicted VO2peak’  ‘aerobic capacity’  ‘physical activity’  ‘physical fitness’  ‘aerobic fitness’ | Humans  Adult  English Language |
| * - searches variant endings to word; $ - xxxx; MeSH – Medical Subject Heading | | | | |
