# Supplementary material for: Outcomes Following eHealth Weight Management Interventions in Adults With Overweight and Obesity From Low Socioeconomic Groups: Protocol for a Systematic Review
Source: JMIR Res Protoc. 2022 Jan 20;11(1):e34546. doi: 10.2196/34546 (PMC8814919; doi:10.2196/34546)
Supplement: Multimedia Appendix 3 [file resprot_v11i1e34546_app3.docx]

**Multimedia Appendix- Database Search Overview**

**Table 1.** Overview of search results

| Database | Date searched | Results |
| --- | --- | --- |
| CINAHL Plus with Full Text (Ebsco) | 06/05/2021 | 218 |
| Embase (Elsevier) | 06/05/2021 | 989 |
| EMCARE (Elievier) | 06/05/2021 | 425 |
| Ovid MEDLINE(R) and Epub Ahead of Print, In-Process & Other Non-Indexed Citations, Daily and Versions(R) 1946 to May 05, 2021 | 06/05/2021 | 624 |
| Total | | **2256** |
| After duplicates removed | | **1545** |

We searched the databases listed above on 6^th^ May 2021. Records from each database were exported to EndNote. Duplicates were removed using hand searching and the “Find Duplicates” function within EndNote. Unique results were uploaded to Rayyan. Example search strategies from each of the bibliographic databases are available below.

Ovid MEDLINE(R), and Epub Ahead of Print, In-Process, In-Data-Review & Other Non-Indexed Citations and Daily <1946 to May 05, 2021>

**Table 2.** Search strategy of MEDLINE database carried out on 6th May 2021

| # | Query | No. Results |
| --- | --- | --- |
| 1 | exp Obesity/ | 224,286 |
| 2 | exp Overweight/ | 231,043 |
| 3 | exp Adult/ | 7,442,878 |
| 4 | (exp Social Class/ or exp Socioeconomic Factors/ or exp Health Status Disparities/) | 474,908 |
| 5 | (exp Health Status Disparities/ or exp Health Services Accessibility/) | 131,230 |
| 6 | exp Poverty/ | 45,299 |
| 7 | exp Income/ | 65,099 |
| 8 | exp Educational Status/ | 53,562 |
| 9 | exp Employment/ | 90,034 |
| 10 | [Obesity.mp.](http://scanmail.trustwave.com/?c=8248&d=8fKT4FVQWOsB-7Q3h5uJUXt6B3wk-AtVg5RfWHFwgQ&u=http%3a%2f%2fObesity%2emp) | 346,052 |
| 11 | [obese.mp.](http://scanmail.trustwave.com/?c=8248&d=8fKT4FVQWOsB-7Q3h5uJUXt6B3wk-AtVg5xdDXZ3hA&u=http%3a%2f%2fobese%2emp) | 135,462 |
| 12 | [overweight.mp.](http://scanmail.trustwave.com/?c=8248&d=8fKT4FVQWOsB-7Q3h5uJUXt6B3wk-AtVg8hUCHcghA&u=http%3a%2f%2foverweight%2emp) | 79,302 |
| 13 | [adult.mp.](http://scanmail.trustwave.com/?c=8248&d=8fKT4FVQWOsB-7Q3h5uJUXt6B3wk-AtVg8kLWyFyhg&u=http%3a%2f%2fadult%2emp) | 5,789,631 |
| 14 | "Social disparit$".mp. | 636 |
| 15 | "Social inequalit$".mp. | 4,047 |
| 16 | "Social inequity".mp. | 166 |
| 17 | "Economic disparity".mp. | 99 |
| 18 | "Economic inequality".mp. | 579 |
| 19 | "Economic inequity".mp. | 43 |
| 20 | "Socio-economic disparity".mp. | 31 |
| 21 | "Socioeconomic disparity".mp. | 168 |
| 22 | "Socioeconomic inequality".mp. | 650 |
| 23 | "Socio-economic inequality".mp. | 141 |
| 24 | "Socioeconomic inequity".mp. | 58 |
| 25 | "Socio-economic inequity".mp. | 18 |
| 26 | "Low income".mp. | 38,756 |
| 27 | "Low education".mp. | 4,293 |
| 28 | [Employment.mp.](http://scanmail.trustwave.com/?c=8248&d=8fKT4FVQWOsB-7Q3h5uJUXt6B3wk-AtVg8lUX3Jz0g&u=http%3a%2f%2fEmployment%2emp) | 93,387 |
| 29 | 1 or 2 or 10 or 11 or 12 | 393,151 |
| 30 | 3 or 13 | 7,917,894 |
| 31 | 4 or 5 or 6 or 7 or 8 or 9 or 14 or 15 or 16 or 17 or 18 or 19 or 20 or 21 or 22 or 23 or 24 or 25 or 26 or 27 or 28 | 627,216 |
| 32 | exp Telemedicine/ | 34,082 |
| 33 | "electronic health".mp. | 34,562 |
| 34 | [telemedicine.mp.](http://scanmail.trustwave.com/?c=8248&d=8fKT4FVQWOsB-7Q3h5uJUXt6B3wk-AtVg5peCXIg0Q&u=http%3a%2f%2ftelemedicine%2emp) | 35,212 |
| 35 | [telehealth.mp.](http://scanmail.trustwave.com/?c=8248&d=8fKT4FVQWOsB-7Q3h5uJUXt6B3wk-AtVg50IWCxxhw&u=http%3a%2f%2ftelehealth%2emp) | 7,344 |
| 36 | [mhealth.mp.](http://scanmail.trustwave.com/?c=8248&d=8fKT4FVQWOsB-7Q3h5uJUXt6B3wk-AtVg5wPX3Fw0g&u=http%3a%2f%2fmhealth%2emp) | 5,842 |
| 37 | [m-health.mp.](http://scanmail.trustwave.com/?c=8248&d=8fKT4FVQWOsB-7Q3h5uJUXt6B3wk-AtVg5oPAiMigQ&u=http%3a%2f%2fm-health%2emp) | 648 |
| 38 | "mobile health".mp. | 8,990 |
| 39 | "interactive media".mp. | 146 |
| 40 | exp Telephone/ | 29,294 |
| 41 | [telephone.mp.](http://scanmail.trustwave.com/?c=8248&d=8fKT4FVQWOsB-7Q3h5uJUXt6B3wk-AtVg5RaXyZ-1A&u=http%3a%2f%2ftelephone%2emp) | 65,271 |
| 42 | [telephone-based.mp.](http://scanmail.trustwave.com/?c=8248&d=8fKT4FVQWOsB-7Q3h5uJUXt6B3wk-AtVg8ldXCAn0w&u=http%3a%2f%2ftelephone-based%2emp) | 1,925 |
| 43 | [phone-based.mp.](http://scanmail.trustwave.com/?c=8248&d=8fKT4FVQWOsB-7Q3h5uJUXt6B3wk-AtVg5hdDnIjgA&u=http%3a%2f%2fphone-based%2emp) | 1,252 |
| 44 | (exp Internet Access/ or (exp Internet/) | 84,525 |
| 45 | [internet.mp.](http://scanmail.trustwave.com/?c=8248&d=8fKT4FVQWOsB-7Q3h5uJUXt6B3wk-AtVg81dAyN21g&u=http%3a%2f%2finternet%2emp) | 110,792 |
| 46 | [internet-based.mp.](http://scanmail.trustwave.com/?c=8248&d=8fKT4FVQWOsB-7Q3h5uJUXt6B3wk-AtVg54OCyMk0w&u=http%3a%2f%2finternet-based%2emp) | 9,577 |
| 47 | exp Web Browser/ | 1,243 |
| 48 | [web.mp.](http://scanmail.trustwave.com/?c=8248&d=8fKT4FVQWOsB-7Q3h5uJUXt6B3wk-AtVg85YXydzgA&u=http%3a%2f%2fweb%2emp) | 131,549 |
| 49 | [web-based.mp.](http://scanmail.trustwave.com/?c=8248&d=8fKT4FVQWOsB-7Q3h5uJUXt6B3wk-AtVg5RfXiAi2w&u=http%3a%2f%2fweb-based%2emp) | 33,806 |
| 50 | (exp Information Dissemination/ or exp Social Media/) | 26,677 |
| 51 | [website.mp.](http://scanmail.trustwave.com/?c=8248&d=8fKT4FVQWOsB-7Q3h5uJUXt6B3wk-AtVg8lZCCcggQ&u=http%3a%2f%2fwebsite%2emp) | 19,571 |
| 52 | [website-based.mp.](http://scanmail.trustwave.com/?c=8248&d=8fKT4FVQWOsB-7Q3h5uJUXt6B3wk-AtVg5hbWyAggw&u=http%3a%2f%2fwebsite-based%2emp) | 100 |
| 53 | exp Electronic Mail/ | 2,763 |
| 54 | [e-mail.mp.](http://scanmail.trustwave.com/?c=8248&d=8fKT4FVQWOsB-7Q3h5uJUXt6B3wk-AtVg85dDHB_1w&u=http%3a%2f%2fe-mail%2emp) | 7,255 |
| 55 | exp Telecommunications/ | 104,449 |
| 56 | "electronic mail".mp. | 3,460 |
| 57 | exp Computers/ | 80,018 |
| 58 | [computers.mp.](http://scanmail.trustwave.com/?c=8248&d=8fKT4FVQWOsB-7Q3h5uJUXt6B3wk-AtVg8hUAid20A&u=http%3a%2f%2fcomputers%2emp) | 73,398 |
| 59 | [computer.mp.](http://scanmail.trustwave.com/?c=8248&d=8fKT4FVQWOsB-7Q3h5uJUXt6B3wk-AtVg5VeXnJ1hA&u=http%3a%2f%2fcomputer%2emp) | 725,533 |
| 60 | [computer-based.mp.](http://scanmail.trustwave.com/?c=8248&d=8fKT4FVQWOsB-7Q3h5uJUXt6B3wk-AtVg5QMW3d0gA&u=http%3a%2f%2fcomputer-based%2emp) | 14,357 |
| 61 | exp Wireless Technology/ | 3,846 |
| 62 | [wireless.mp.](http://scanmail.trustwave.com/?c=8248&d=8fKT4FVQWOsB-7Q3h5uJUXt6B3wk-AtVg5kLCSF3gA&u=http%3a%2f%2fwireless%2emp) | 16,807 |
| 63 | exp Cell Phone/ | 17,091 |
| 64 | "mobile phone".mp. | 7,823 |
| 65 | "cell phone".mp. | 10,377 |
| 66 | "cellular phone".mp. | 565 |
| 67 | exp Smartphone/ | 5,741 |
| 68 | [smartphone.mp.](http://scanmail.trustwave.com/?c=8248&d=8fKT4FVQWOsB-7Q3h5uJUXt6B3wk-AtVg5QJCiVx0Q&u=http%3a%2f%2fsmartphone%2emp) | 13,667 |
| 69 | exp Computers, Handheld/ | 9,391 |
| 70 | "mobile device".mp. | 1,339 |
| 71 | "personal digital assistant".mp. | 580 |
| 72 | [pda.mp.](http://scanmail.trustwave.com/?c=8248&d=8fKT4FVQWOsB-7Q3h5uJUXt6B3wk-AtVg85dAnAl0A&u=http%3a%2f%2fpda%2emp) | 13,097 |
| 73 | "interactive voice response".mp. | 851 |
| 74 | [ivr.mp.](http://scanmail.trustwave.com/?c=8248&d=8fKT4FVQWOsB-7Q3h5uJUXt6B3wk-AtVg81eCC11gQ&u=http%3a%2f%2fivr%2emp) | 1,569 |
| 75 | exp Text Messaging/ | 3,396 |
| 76 | "text message".mp. | 1,817 |
| 77 | "text messaging".mp. | 4,680 |
| 78 | [SMS.mp.](http://scanmail.trustwave.com/?c=8248&d=8fKT4FVQWOsB-7Q3h5uJUXt6B3wk-AtVg51ZDiFz1g&u=http%3a%2f%2fSMS%2emp) | 6,547 |
| 79 | [bluetooth.mp.](http://scanmail.trustwave.com/?c=8248&d=8fKT4FVQWOsB-7Q3h5uJUXt6B3wk-AtVg5lZWSZxhw&u=http%3a%2f%2fbluetooth%2emp) | 1,371 |
| 80 | [chat.mp.](http://scanmail.trustwave.com/?c=8248&d=8fKT4FVQWOsB-7Q3h5uJUXt6B3wk-AtVg8hbCSV-0A&u=http%3a%2f%2fchat%2emp) | 8,641 |
| 81 | "chat room".mp. | 140 |
| 82 | "instant message".mp. | 22 |
| 83 | [IM.mp.](http://scanmail.trustwave.com/?c=8248&d=8fKT4FVQWOsB-7Q3h5uJUXt6B3wk-AtVg55bCicggA&u=http%3a%2f%2fIM%2emp) | 117,272 |
| 84 | exp Social Media/ | 9,910 |
| 85 | [twitter.mp.](http://scanmail.trustwave.com/?c=8248&d=8fKT4FVQWOsB-7Q3h5uJUXt6B3wk-AtVg5tdCHFy2w&u=http%3a%2f%2ftwitter%2emp) | 4,020 |
| 86 | [tweet.mp.](http://scanmail.trustwave.com/?c=8248&d=8fKT4FVQWOsB-7Q3h5uJUXt6B3wk-AtVg5VdCyUg0g&u=http%3a%2f%2ftweet%2emp) | 574 |
| 87 | exp Blogging/ | 1,016 |
| 88 | [blog.mp.](http://scanmail.trustwave.com/?c=8248&d=8fKT4FVQWOsB-7Q3h5uJUXt6B3wk-AtVg5xdCCIk0A&u=http%3a%2f%2fblog%2emp) | 872 |
| 89 | exp Social Networking/ | 4,498 |
| 90 | "social network".mp. | 10,338 |
| 91 | [tailored.mp.](http://scanmail.trustwave.com/?c=8248&d=8fKT4FVQWOsB-7Q3h5uJUXt6B3wk-AtVg5kIC3J02g&u=http%3a%2f%2ftailored%2emp) | 67,223 |
| 92 | [automated.mp.](http://scanmail.trustwave.com/?c=8248&d=8fKT4FVQWOsB-7Q3h5uJUXt6B3wk-AtVg5ReDCd2hw&u=http%3a%2f%2fautomated%2emp) | 146,962 |
| 93 | "individualized programme".mp. | 18 |
| 94 | "individualised programme".mp. | 17 |
| 95 | (exp Remote Consultation/ or [remote.mp.](http://scanmail.trustwave.com/?c=8248&d=8fKT4FVQWOsB-7Q3h5uJUXt6B3wk-AtVg5xZCyJz0g&u=http%3a%2f%2fremote%2emp)) | 82,708 |
| 96 | [self-monitoring.mp.](http://scanmail.trustwave.com/?c=8248&d=8fKT4FVQWOsB-7Q3h5uJUXt6B3wk-AtVg55ZCHcj0w&u=http%3a%2f%2fself-monitoring%2emp) | 13,501 |
| 97 | (exp Feedback/ or [feedback.mp.](http://scanmail.trustwave.com/?c=8248&d=8fKT4FVQWOsB-7Q3h5uJUXt6B3wk-AtVg80MDHd20A&u=http%3a%2f%2ffeedback%2emp)) | 164,916 |
| 98 | [prompt.mp.](http://scanmail.trustwave.com/?c=8248&d=8fKT4FVQWOsB-7Q3h5uJUXt6B3wk-AtVg8hVDiNygA&u=http%3a%2f%2fprompt%2emp) | 68,323 |
| 99 | [reminder.mp.](http://scanmail.trustwave.com/?c=8248&d=8fKT4FVQWOsB-7Q3h5uJUXt6B3wk-AtVg8pVWCd_hA&u=http%3a%2f%2freminder%2emp) | 10,349 |
| 100 | 32 or 33 or 34 or 35 or 36 or 37 or 38 or 39 or 40 or 41 or 42 or 43 or 44 or 45 or 46 or 47 or 48 or 49 or 50 or 51 or 52 or 53 or 54 or 55 or 56 or 57 or 58 or 59 or 60 or 61 or 62 or 63 or 64 or 65 or 66 or 67 or 68 or 69 or 70 or 71 or 72 or 73 or 74 or 75 or 76 or 77 or 78 or 79 or 80 or 81 or 82 or 83 or 84 or 85 or 86 or 87 or 88 or 89 or 90 or 91 or 92 or 93 or 94 or 95 or 96 or 97 or 98 or 99 | 1,744,879 |
| 101 | exp Weight Loss/ | 43,832 |
| 102 | "weight loss".mp. | 104,711 |
| 103 | "weight reduction".mp. | 11,824 |
| 104 | (exp Weight Reduction Programs/ or "weight reduction program*".mp.) | 3,193 |
| 105 | "weight loss maintenance".mp. | 928 |
| 106 | (exp Weight Gain/ or "weight gain prevention".mp.) | 33,062 |
| 107 | "obesity trials".mp. | 25 |
| 108 | "obesity reduction".mp. | 195 |
| 109 | "obesity prevention".mp. | 4,278 |
| 110 | ("Body mass index".mp. or exp Body Mass Index/) | 255,349 |
| 111 | "BMI".mp. | 157,476 |
| 112 | exp Cardiorespiratory Fitness/ | 2,072 |
| 113 | (exp Physical Fitness/ or "cardiopulmonary fitness".mp.) | 32,366 |
| 114 | "cardiovascular fitness".mp. | 1,418 |
| 115 | [VO2max.mp.](http://scanmail.trustwave.com/?c=8248&d=8fKT4FVQWOsB-7Q3h5uJUXt6B3wk-AtVg5gJCid32g&u=http%3a%2f%2fVO2max%2emp) | 9,246 |
| 116 | "estimated VO2max".mp. | 180 |
| 117 | "predicted VO2peak".mp. | 50 |
| 118 | (exp Exercise Tolerance/ or "aerobic capacity".mp.) | 18,115 |
| 119 | ("physical activity".mp. or exp Exercise/) | 281,548 |
| 120 | “physical fitness”.mp. | 32,928 |
| 121 | “aerobic fitness”.mp. | 3,375 |
| 122 | 101 or 102 or 103 or 104 or 105 or 106 or 107 or 108 or 109 or 110 or 111 or 112 or 113 or 114 or 115 or 116 or 117 or 118 or 119 or 120 or 121 | 686,745 |
| 123 | “weight loss program*”.mp. | 2,448 |
| 124 | exp Weight Reduction Programs/ | 2,547 |
| 125 | ([diet.mp.](http://scanmail.trustwave.com/?c=8248&d=8fKT4FVQWOsB-7Q3h5uJUXt6B3wk-AtVg5VaDy10hw&u=http%3a%2f%2fdiet%2emp) or exp Diet, Healthy/ or exp "Diet, Food, and Nutrition"/ or exp Diet/ or exp Diet, Ketogenic/ or exp Diet, High-Protein Low-Carbohydrate/ or exp Diet, Carbohydrate-Restricted/) | 1,923,039 |
| 126 | [nutrition.mp.](http://scanmail.trustwave.com/?c=8248&d=8fKT4FVQWOsB-7Q3h5uJUXt6B3wk-AtVg5oID3Bz0A&u=http%3a%2f%2fnutrition%2emp) | 242,809 |
| 127 | ("Physical activity".mp. or exp Exercise/) | 281,548 |
| 128 | [exercise.mp.](http://scanmail.trustwave.com/?c=8248&d=8fKT4FVQWOsB-7Q3h5uJUXt6B3wk-AtVg8lUCnUkhg&u=http%3a%2f%2fexercise%2emp) | 371,854 |
| 129 | ("weight management program*".mp. or exp Weight Reduction Programs/) | 3,503 |
| 130 | "weight management intervention".mp. | 278 |
| 131 | (exp Weight Loss/ or "weight loss intervention".mp.) | 44,361 |
| 132 | (exp Health Behavior/ or "behaviour change".mp. or exp Behavior Therapy/) | 408,345 |
| 133 | "behaviour change techniques".mp. | 644 |
| 134 | ("Low calorie diet".mp. or exp Caloric Restriction/) | 7,571 |
| 135 | "very low calorie ketogenic diet".mp. | 50 |
| 136 | 123 or 124 or 125 or 126 or 127 or 128 or 129 or 130 or 131 or 132 or 133 or 134 or 135 | 2,821,267 |
| 137 | 29 and 30 and 31 and 100 and 122 and 136 | 649 |
| 138 | 137 | 649 |
| 139 | limit 137 to (english language and humans) | 624 |
| “” – literal string; / - limits a subject heading to a known subheading; * - searches variant endings to word; mp - limits to title, abstract, subject heading and other fields | | |

**Table 3.** Search strategy of EBSCO database carried out on 6th May 2021

| # | Query | No. Results |
| --- | --- | --- |
| S110 | S4 AND S105 AND S106 AND S107 AND S108 AND S109 | 218 |
| S109 | S85 OR S86 OR S87 OR S88 OR S89 OR S90 OR S91 OR S92 OR S93 OR S94 OR S95 OR S96 OR S97 OR S98 OR S99 OR S100 OR S101 OR S102 OR S103 OR S104 | 260,977 |
| S108 | S73 OR S74 OR S75 OR S76 OR S77 OR S78 OR S79 OR S80 OR S81 OR S82 OR S83 OR S84 | 383,086 |
| S107 | S20 OR S21 OR S22 OR S23 OR S24 OR S25 OR S26 OR S27 OR S28 OR S29 OR S30 OR S31 OR S32 OR S33 OR S34 OR S35 OR S36 OR S37 OR S38 OR S39 OR S40 OR S41 OR S42 OR S43 OR S44 OR S45 OR S46 OR S47 OR S48 OR S49 OR S50 OR S51 OR S52 OR S53 OR S54 OR S55 OR S56 OR S57 OR S58 OR S59 OR S60 OR S61 OR S62 OR S63 OR S64 OR S65 OR S66 OR S67 OR S68 OR S69 OR S70 OR S71 OR S72 | 571,502 |
| S106 | S5 OR S6 OR S7 OR S8 OR S9 OR S10 OR S11 OR S12 OR S13 OR S14 OR S15 OR S16 OR S17 OR S18 OR S19 | 198,728 |
| S105 | S1 OR S2 OR S3 | 145,120 |
| S104 | "aerobic fitness" | 1,560 |
| S103 | "physical fitness" | 19,883 |
| S102 | "physical activity" | 101,171 |
| S101 | "aerobic capacity" | 3,597 |
| S100 | "predicted VO2peak" | 19 |
| S99 | "estimated VO2max" | 73 |
| S98 | VO2max | 2,704 |
| S97 | "cardiovascular fitness" | 729 |
| S96 | "cardiopulmonary fitness" | 153 |
| S95 | "cardiorespiratory fitness" | 3,280 |
| S94 | "obesity prevention" | 17,197 |
| S93 | "obesity reduction" | 87 |
| S92 | "obesity trials" | 5 |
| S91 | "weight gain prevention" | 894 |
| S90 | "weight loss maintenance" | 483 |
| S89 | "weight reduction" | 14,332 |
| S88 | "weight reduction program*" | 3,115 |
| S87 | (MH "Weight Reduction Programs") OR (MH "Weight Loss") | 24,985 |
| S86 | BMI | 58,892 |
| S85 | (MH "Body Mass Index") OR (MH "Fat Free Mass") OR ""Body mass index"" | 120,760 |
| S84 | “very low calorie ketogenic diet” | 15 |
| S83 | “Low calorie diet” | 2,678 |
| S82 | “behaviour change techniques” | 409 |
| S81 | “behaviour change” | 3,490 |
| S80 | “weight loss intervention” | 700 |
| S79 | “weight management intervention” | 175 |
| S78 | “weight management program*” | 798 |
| S77 | (MH "Exercise") OR "Exercise" OR (MH "Teaching: Prescribed Activity-Exercise (Iowa NIC)") OR (MH "Aerobic Exercises") OR (MH "Exercise Promotion (Iowa NIC)") | 177,838 |
| S76 | (MH "Physical Activity") OR "“Physical activity”" | 102,472 |
| S75 | (MH "Nutrition") | 28,755 |
| S74 | (MH "Diet, Fat-Restricted") OR (MH "Diet, Low Carbohydrate") OR (MH "Restricted Diet") OR "Diet" | 142,020 |
| S73 | (MH "Weight Reduction Programs") OR (MH "Weight Reduction Assistance (Iowa NIC)") OR (MH "Weight Loss") OR "“weight loss program*”" | 25,297 |
| S72 | reminder | 8,434 |
| S71 | prompt | 19,027 |
| S70 | feedback | 44,435 |
| S69 | "self-monitoring" | 6,749 |
| S68 | remote | 19,070 |
| S67 | "individualised programme" | 13 |
| S66 | "individualized programme" | 9 |
| S65 | automated | 21,728 |
| S64 | tailored | 21,812 |
| S63 | "social network" | 9,739 |
| S62 | blog | 5,288 |
| S61 | tweet | 2,039 |
| S60 | twitter | 3,528 |
| S59 | IM | 14,035 |
| S58 | "instant message" | 15 |
| S57 | "chat room" | 112 |
| S56 | chat | 1,910 |
| S55 | bluetooth | 365 |
| S54 | SMS | 1,674 |
| S53 | "text messaging" | 3,938 |
| S52 | "text message" | 2,539 |
| S51 | ivr | 411 |
| S50 | "interactive voice response" | 1,260 |
| S49 | pda | 4,977 |
| S48 | "personal digital assistant" | 2,688 |
| S47 | "mobile device" | 739 |
| S46 | smartphone | 8,049 |
| S45 | "cellular phone" | 2,133 |
| S44 | "cell phone" | 2,173 |
| S43 | "mobile phone" | 3,470 |
| S42 | wireless | 14,362 |
| S41 | "computer-based" | 4,623 |
| S40 | computer | 146,014 |
| S39 | computers | 146,014 |
| S38 | "e-mail-based" | 52 |
| S37 | "electronic mail" | 2,414 |
| S36 | "website-based" | 42 |
| S35 | website | 23,030 |
| S34 | "web-based" | 16,001 |
| S33 | web | 133,908 |
| S32 | "internet-based" | 4,788 |
| S31 | "internet" | 69,734 |
| S30 | "phone-based" | 574 |
| S29 | "telephone-based" | 1,170 |
| S28 | "telephone" | 42,039 |
| S27 | "interactive media" | 194 |
| S26 | "mobile health" | 19,200 |
| S25 | "m-health" | 216 |
| S24 | "mhealth" | 16,972 |
| S23 | telehealth | 21,251 |
| S22 | (MH "Telemedicine+") OR (MH "Telerehabilitation") OR (MH "Telehealth") OR ""telemedicine"" | 26,990 |
| S21 | ""electronic health"" | 34,378 |
| S20 | "e-health" | 1,489 |
| S19 | "Employment" | 68,291 |
| S18 | "Low education" | 1,948 |
| S17 | "Low income" | 20,727 |
| S16 | "Socio-economic inequity" | 3 |
| S15 | "Socioeconomic inequity" | 22 |
| S14 | "Socio-economic inequality" | 72 |
| S13 | "Socioeconomic inequality" | 311 |
| S12 | ""Socioeconomic disparity"" | 962 |
| S11 | ""Socio-economic disparity"" | 121 |
| S10 | ""Economic inequity"" | 69 |
| S9 | ""Economic inequality"" | 689 |
| S8 | (MH "Healthcare Disparities") OR (MH "Health Status Disparities") OR (MH "Economic Status") OR ""Economic disparity"" OR (MH "Economic Factors") OR (MH "Socioeconomic Factors") | 110,151 |
| S7 | ""Social inequity"" | 291 |
| S6 | "Social inequalit*" | 1,819 |
| S5 | ""Social disparit*"" OR (MH "Social Class+") OR (MH "Social Support Index") | 13,362 |
| S4 | Adult | 1,422,495 |
| S3 | "Overweight" | 73,109 |
| S2 | "obese" | 41,687 |
| S1 | (MH "Obesity+") OR "Obesity" OR (MH "Obesity, Morbid") | 133,100 |
