# Supplementary material for: Outcomes Following eHealth Weight Management Interventions in Adults With Overweight and Obesity From Low Socioeconomic Groups: Protocol for a Systematic Review
Source: JMIR Res Protoc. 2022 Jan 20;11(1):e34546. doi: 10.2196/34546 (PMC8814919; doi:10.2196/34546)
Supplement: Multimedia Appendix 4 [file resprot_v11i1e34546_app4.docx]

**Multimedia Appendix- Risk of Bias Assessment Tools**

The criteria outlined below are based on the Joanna Briggs Institute Critical Appraisal Tool’s (Moola, et al., 2020). Rates will assess study bias by independently scoring studies on each of the criteria based on the descriptors. High risk = 2, low risk = 0, unsure = 1) with the total score a summation of the criteria ratings.

**Table 1.** Joanna Briggs Institute critical appraisal checklist for analytical cross sectional studies with descriptors and scoring.

| Criterion | Description | Score |
| --- | --- | --- |
| Were the criteria for inclusion in the sample clearly defined? | The authors should provide clear inclusion and exclusion criteria that they developed prior to recruitment of the study participants. The inclusion/exclusion criteria should be specified (e.g., risk, stage of disease progression) with sufficient detail and all the necessary information critical to the study. | /2 |
| Were the study subjects and the setting described in detail? | The study sample should be described in sufficient detail so that other researchers can determine if it is comparable to the population of interest to them. The authors should provide a clear description of the population from which the study participants were selected or recruited, including demographics, location, and time period. | /2 |
| Was the exposure measured in a valid and reliable way? | The study should clearly describe the method of measurement of exposure. Assessing validity requires that a 'gold standard' is available to which the measure can be compared. The validity of exposure measurement usually relates to whether a current measure is appropriate or whether a measure of past exposure is needed.  Reliability refers to the processes included in an epidemiological study to check repeatability of measurements of the exposures. These usually include intra-observer reliability and inter-observer reliability. | /2 |
| Were objective, standard criteria used for measurement of the condition? | It is useful to determine if patients were included in the study based on either a specified diagnosis or definition. This is more likely to decrease the risk of bias. Characteristics are another useful approach to matching groups, and studies that did not use specified diagnostic methods or definitions should provide evidence on matching by key characteristics. | /2 |
| Were confounding factors identified? | Confounding has occurred where the estimated intervention exposure effect is biased by the presence of some difference between the comparison groups (apart from the exposure investigated/of interest). Typical confounders include baseline characteristics, prognostic factors, or concomitant exposures (e.g. smoking). A confounder is a difference between the comparison groups and it influences the direction of the study results. A high quality study at the level of cohort design will identify the potential confounders and measure them (where possible). This is difficult for studies where behavioral, attitudinal or lifestyle factors may impact on the results. | /2 |
| Were strategies to deal with confounding factors stated? | Strategies to deal with effects of confounding factors may be dealt within the study design or in data analysis. By matching or stratifying sampling of participants, effects of confounding factors can be adjusted for. When dealing with adjustment in data analysis, assess the statistics used in the study. Most will be some form of multivariate regression analysis to account for the confounding factors measured. | /2 |
| Were the outcomes measured in a valid and reliable way? | Read the methods section of the paper. If for e.g. lung cancer is assessed based on existing definitions or diagnostic criteria, then the answer to this question is likely to be yes. If lung cancer is assessed using observer reported, or self-reported scales, the risk of over- or under-reporting is increased, and objectivity is compromised. Importantly, determine if the measurement tools used were validated instruments as this has a significant impact on outcome assessment validity.  Having established the objectivity of the outcome measurement (e.g. lung cancer) instrument, it’s important to establish how the measurement was conducted. Were those involved in collecting data trained or educated in the use of the instrument/s? (e.g. radiographers). If there was more than one data collector, were they similar in terms of level of education, clinical or research experience, or level of responsibility in the piece of research being appraised? | /2 |
| Was appropriate statistical analysis used? | As with any consideration of statistical analysis, consideration should be given to whether there was a more appropriate alternate statistical method that could have been used. The methods section should be detailed enough for reviewers to identify which analytical techniques were used (in particular, regression or stratification) and how specific confounders were measured.  For studies utilizing regression analysis, it is useful to identify if the study identified which variables were included and how they related to the outcome. If stratification was the analytical approach used, were the strata of analysis defined by the specified variables? Additionally, it is also important to assess the appropriateness of the analytical strategy in terms of the assumptions associated with the approach as differing methods of analysis are based on differing assumptions about the data and how it will respond. | /2 |
|  | Total score | /16 |

**Table 2.** Joanna Briggs Institute critical appraisal checklist for case control studies with descriptors and scoring.

| Criterion | Description | Score |
| --- | --- | --- |
| Were the groups comparable other than the presence of disease in cases or the absence of disease in controls? | The control group should be representative of the source population that produced the cases. This is usually done by individual matching; wherein controls are selected for each case on the basis of similarity with respect to certain characteristics other than the exposure of interest. Frequency or group matching is an alternative method. Selection bias may result if the groups are not comparable. | /2 |
| Were cases and controls matched appropriately? | As in item 1, the study should include clear definitions of the source population. Sources from which cases and controls were recruited should be carefully looked at. For example, cancer registries may be used to recruit participants in a study examining risk factors for lung cancer, which typify population-based case control studies. Study participants may be selected from the target population, the source population, or from a pool of eligible participants (such as in hospital-based case control studies). | /2 |
| Were the same criteria used for identification of cases and controls? | It is useful to determine if patients were included in the study based on either a specified diagnosis or definition. This is more likely to decrease the risk of bias. Characteristics are another useful approach to matching groups, and studies that did not use specified diagnostic methods or definitions should provide evidence on matching by key characteristics. A case should be defined clearly. It is also important that controls must fulfil all the eligibility criteria defined for the cases except for those relating to diagnosis of the disease. | /2 |
| Was exposure measured in a standard, valid and reliable way? | The study should clearly describe the method of measurement of exposure. Assessing validity requires that a 'gold standard' is available to which the measure can be compared. The validity of exposure measurement usually relates to whether a current measure is appropriate or whether a measure of past exposure is needed.  Case control studies may investigate many different ‘exposures’ that may or may not be associated with the condition. In these cases, reviewers should use the main exposure of interest for their review to answer this question when using this tool at the study level.  Reliability refers to the processes included in an epidemiological study to check repeatability of measurements of the exposures. These usually include intra-observer reliability and inter-observer reliability. | /2 |
| Was exposure measured in the same way for cases and controls? | As in item 4, the study should clearly describe the method of measurement of exposure. The exposure measures should be clearly defined and described in detail. Assessment of exposure or risk factors should have been carried out according to same procedures or protocols for both cases and controls. | /2 |
| Were confounding factors identified? | Confounding has occurred where the estimated intervention exposure effect is biased by the presence of some difference between the comparison groups (apart from the exposure investigated/of interest). Typical confounders include baseline characteristics, prognostic factors, or concomitant exposures (e.g. smoking). A confounder is a difference between the comparison groups and it influences the direction of the study results. A high quality study at the level of case control design will identify the potential confounders and measure them (where possible). This is difficult for studies where behavioral, attitudinal or lifestyle factors may impact on the results. | /2 |
| Were strategies to deal with confounding factors stated? | Strategies to deal with effects of confounding factors may be dealt within the study design or in data analysis. By matching or stratifying sampling of participants, effects of confounding factors can be adjusted for. When dealing with adjustment in data analysis, assess the statistics used in the study. Most will be some form of multivariate regression analysis to account for the confounding factors measured. Look out for a description of statistical methods as regression methods such as logistic regression are usually employed to deal with confounding factors/ variables of interest. | /2 |
| Were outcomes assessed in a standard, valid and reliable way for cases and controls? | Read the methods section of the paper. If for e.g. lung cancer is assessed based on existing definitions or diagnostic criteria, then the answer to this question is likely to be yes. If lung cancer is assessed using observer reported, or self-reported scales, the risk of over- or under-reporting is increased, and objectivity is compromised. Importantly, determine if the measurement tools used were validated instruments as this has a significant impact on outcome assessment validity.  Having established the objectivity of the outcome measurement (e.g. lung cancer) instrument, it’s important to establish how the measurement was conducted. Were those involved in collecting data trained or educated in the use of the instrument/s? (e.g. radiographers). If there was more than one data collector, were they similar in terms of level of education, clinical or research experience, or level of responsibility in the piece of research being appraised? | /2 |
| Was the exposure period of interest long enough to be meaningful? | It is particularly important in a case control study that the exposure time was sufficient enough to show an association between the exposure and the outcome. It may be that the exposure period may be too short or too long to influence the outcome. | /2 |
| Was appropriate statistical analysis used? | As with any consideration of statistical analysis, consideration should be given to whether there was a more appropriate alternate statistical method that could have been used. The methods section should be detailed enough for reviewers to identify which analytical techniques were used (in particular, regression or stratification) and how specific confounders were measured.  For studies utilizing regression analysis, it is useful to identify if the study identified which variables were included and how they related to the outcome. If stratification was the analytical approach used, were the strata of analysis defined by the specified variables? Additionally, it is also important to assess the appropriateness of the analytical strategy in terms of the assumptions associated with the approach as differing methods of analysis are based on differing assumptions about the data and how it will respond. | /2 |
|  | Total score | /20 |

**Table 3.** Joanna Briggs Institute critical appraisal checklist for cohort studies with descriptors and scoring.

| Criterion | Description | Score |
| --- | --- | --- |
| Were the two groups similar and recruited from the same population? | Check the paper carefully for descriptions of participants to determine if patients within and across groups have similar characteristics in relation to exposure (e.g. risk factor under investigation). The two groups selected for comparison should be as similar as possible in all characteristics except for their exposure status, relevant to the study in question. The authors should provide clear inclusion and exclusion criteria that they developed prior to recruitment of the study participants. | /2 |
| Were the exposures measured similarly to assign people to both exposed and unexposed groups? | A high quality study at the level of cohort design should mention or describe how the exposures were measured. The exposure measures should be clearly defined and described in detail. This will enable reviewers to assess whether or not the participants received the exposure of interest. | /2 |
| Was the exposure measured in a valid and reliable way? | The study should clearly describe the method of measurement of exposure. Assessing validity requires that a 'gold standard' is available to which the measure can be compared. The validity of exposure measurement usually relates to whether a current measure is appropriate or whether a measure of past exposure is needed.  Reliability refers to the processes included in an epidemiological study to check repeatability of measurements of the exposures. These usually include intra-observer reliability and inter-observer reliability. | /2 |
| Were confounding factors identified? | Confounding has occurred where the estimated intervention exposure effect is biased by the presence of some difference between the comparison groups (apart from the exposure investigated/of interest). Typical confounders include baseline characteristics, prognostic factors, or concomitant exposures (e.g. smoking). A confounder is a difference between the comparison groups and it influences the direction of the study results. A high quality study at the level of cohort design will identify the potential confounders and measure them (where possible). This is difficult for studies where behavioral, attitudinal or lifestyle factors may impact on the results. | /2 |
| Were strategies to deal with confounding factors stated? | Strategies to deal with effects of confounding factors may be dealt within the study design or in data analysis. By matching or stratifying sampling of participants, effects of confounding factors can be adjusted for. When dealing with adjustment in data analysis, assess the statistics used in the study. Most will be some form of multivariate regression analysis to account for the confounding factors measured. Look out for a description of statistical methods as regression methods such as logistic regression are usually employed to deal with confounding factors/variables of interest. | /2 |
| Were the groups/participants free of the outcome at the start of the study (or at the moment of exposure)? | The participants should be free of the outcomes of interest at the start of the study. Refer to the ‘methods’ section in the paper for this information, which is usually found in descriptions of participant/sample recruitment, definitions of variables, and/or inclusion/exclusion criteria. | /2 |
| Were the outcomes measured in a valid and reliable way? | Read the methods section of the paper. If for e.g. lung cancer is assessed based on existing definitions or diagnostic criteria, then the answer to this question is likely to be yes. If lung cancer is assessed using observer reported, or self-reported scales, the risk of over- or under-reporting is increased, and objectivity is compromised. Importantly, determine if the measurement tools used were validated instruments as this has a significant impact on outcome assessment validity.  Having established the objectivity of the outcome measurement (e.g. lung cancer) instrument, it’s important to establish how the measurement was conducted. Were those involved in collecting data trained or educated in the use of the instrument/s? (e.g. radiographers). If there was more than one data collector, were they similar in terms of level of education, clinical or research experience, or level of responsibility in the piece of research being appraised? | /2 |
| Was the follow up time reported and sufficient to be long enough for outcomes to occur? | The appropriate length of time for follow up will vary with the nature and characteristics of the population of interest and/or the intervention, disease or exposure. To estimate an appropriate duration of follow up, read across multiple papers and take note of the range for duration of follow up. The opinions of experts in clinical practice or clinical research may also assist in determining an appropriate duration of follow up. For example, a longer timeframe may be needed to examine the association between occupational exposure to asbestos and the risk of lung cancer. It is important, particularly in cohort studies that follow up is long enough to enable the outcomes. However, it should be remembered that the research question and outcomes being examined would probably dictate the follow up time. | /2 |
| Was follow up complete, and if not, were the reasons to loss to follow up described and explored? | It is important in a cohort study that a greater percentage of people are followed up. As a general guideline, at least 80% of patients should be followed up. Generally a dropout rate of 5% or less is considered insignificant. A rate of 20% or greater is considered to significantly impact on the validity of the study. However, in observational studies conducted over a lengthy period of time a higher dropout rate is to be expected. A decision on whether to include or exclude a study because of a high dropout rate is a matter of judgement based on the reasons why people dropped out, and whether dropout rates were comparable in the exposed and unexposed groups.  Reporting of efforts to follow up participants that dropped out may be regarded as an indicator of a well conducted study. Look for clear and justifiable description of why people were left out, excluded, dropped out etc. If there is no clear description or a statement in this regards, this will be a 'No'. | /2 |
| Were strategies to address incomplete follow up utilized? | Some people may withdraw due to change in employment or some may die; however, it is important that their outcomes are assessed. Selection bias may occur as a result of incomplete follow up. Therefore, participants with unequal follow up periods must be taken into account in the analysis, which should be adjusted to allow for differences in length of follow up periods. This is usually done by calculating rates which use person-years at risk, i.e. considering time in the denominator. | /2 |
| Was appropriate statistical analysis used? | As with any consideration of statistical analysis, consideration should be given to whether there was a more appropriate alternate statistical method that could have been used. The methods section of cohort studies should be detailed enough for reviewers to identify which analytical techniques were used (in particular, regression or stratification) and how specific confounders were measured.  For studies utilizing regression analysis, it is useful to identify if the study identified which variables were included and how they related to the outcome. If stratification was the analytical approach used, were the strata of analysis defined by the specified variables? Additionally, it is also important to assess the appropriateness of the analytical strategy in terms of the assumptions associated with the approach as differing methods of analysis are based on differing assumptions about the data and how it will respond. | /2 |
|  | Total score | /22 |

**Table 4.** Joanna Briggs Institute critical appraisal checklist for quasi-experimental studies with descriptors and scoring.

| Criterion | Description | Score |
| --- | --- | --- |
| Is it clear in the study what is the ‘cause’ and what is the ‘effect’ (i.e. there is no confusion about which variable comes first)? | Ambiguity with regards to the temporal relationship of variables constitutes a threat to the internal validity of a study exploring causal relationships. The ‘cause’ (the independent variable, that is, the treatment or intervention of interest) should occur in time before the explored ‘effect’ (the dependent variable, which is the effect or outcome of interest). Check if it is clear which variable is manipulated as a potential cause. Check if it is clear which variable is measured as the effect of the potential cause. Is it clear that the ‘cause’ was manipulated before the occurrence of the ‘effect’? | /2 |
| Were the participants included in any comparisons similar? | The differences between participants included in compared groups constitute a threat to the internal validity of a study exploring causal relationships. If there are differences between participants included in compared groups there is a risk of selection bias. If there are differences between participants included in the compared groups maybe the ‘effect’ cannot be attributed to the potential ‘cause’, as maybe it is plausible that the ‘effect’ may be explained by the differences between participants, that is, by selection bias. Check the characteristics reported for participants. Are the participants from the compared groups similar with regards to the characteristics that may explain the effect even in the absence of the ‘cause’, for example, age, severity of the disease, stage of the disease, co-existing conditions and so on? | /2 |
| Were the participants included in any comparisons receiving similar treatment/care, other than the exposure or intervention of interest? | In order to attribute the ‘effect’ to the ‘cause’ (the exposure or intervention of interest), assuming that there is no selection bias, there should be no other difference between the groups in terms of treatments or care received, other than the manipulated ‘cause’ (the intervention of interest). If there are other exposures or treatments occurring in the same time with the ‘cause’, other than the intervention of interest, then potentially the ‘effect’ cannot be attributed to the intervention of interest, as it is plausible that the ‘effect’ may be explained by other exposures or treatments, other than the intervention of interest, occurring in the same time with the intervention of interest. Check the reported exposures or interventions received by the compared groups. Are there other exposures or treatments occurring in the same time with the intervention of interest? Is it plausible that the ‘effect’ may be explained by other exposures or treatments occurring in the same time with the intervention of interest? | /2 |
| Was there a control group? | Control groups offer the conditions to explore what would have happened with groups exposed to other different treatments, other than to the potential ‘cause’ (the intervention of interest). The comparison of the treated group (the group exposed to the examined ‘cause’, that is, the group receiving the intervention of interest) with such other groups strengthens the examination of the causal plausibility. The validity of causal inferences is strengthened in studies with at least one independent control group compared to studies without an independent control group. Check if there are independent, separate groups, used as control groups in the study. [Note: The control group should be an independent, separate control group, not the pre-test group in a single group pre-test post-test design.] | /2 |
| Were there multiple measurements of the outcome both pre and post the intervention/exposure? | In order to show that there is a change in the outcome (the ‘effect’) as a result of the intervention/treatment (the ‘cause’) it is necessary to compare the results of measurement before and after the intervention/treatment. If there is no measurement before the treatment and only measurement after the treatment is available it is not known if there is a change after the treatment compared to before the treatment. If multiple measurements are collected before the intervention/treatment is implemented then it is possible to explore the plausibility of alternative explanations other than the proposed ‘cause’ (the intervention of interest) for the observed ‘effect’, such as the naturally occurring changes in the absence of the ‘cause’, and changes of high (or low) scores towards less extreme values even in the absence of the ‘cause’ (sometimes called regression to the mean). If multiple measurements are collected after the intervention/treatment is implemented it is possible to explore the changes of the ‘effect’ in time in each group and to compare these changes across the groups. Check if measurements were collected before the intervention of interest was implemented. Were there multiple pre-test measurements? Check if measurements were collected after the intervention of interest was implemented. Were there multiple post-test measurements? | /2 |
| Was follow up complete and if not, were differences between groups in  terms of their follow up adequately described and analyzed? | If there are differences with regards to the loss to follow up between the compared groups these differences represent a threat to the internal validity of a study exploring causal effects as these differences may provide a plausible alternative explanation for the observed ‘effect’ even in the absence of the ‘cause’ (the treatment or exposure of interest). Check if there were differences with regards to the loss to follow up between the compared groups. If follow up was incomplete (that is, there is incomplete information on all participants), examine the reported details about the strategies used in order to address incomplete follow up, such as descriptions of loss to follow up (absolute numbers; proportions; reasons for loss to follow up; patterns of loss to follow up) and impact analyses (the analyses of the impact of loss to follow up on results). Was there a description of the incomplete follow up (number of participants and the specific reasons for loss to follow up)? If there are differences between groups with regards to the loss to follow up, was there an analysis of patterns of loss to follow up? If there are differences between the groups with regards to the loss to follow up, was there an analysis of the impact of the loss to follow up on the results? | /2 |
| Were the outcomes of participants included in any comparisons  measured in the same way? | If the outcome (the ‘effect’) is not measured in the same way in the compared groups there is a threat to the internal validity of a study exploring a causal relationship as the differences in outcome measurements may be confused with an effect of the treatment or intervention of interest (the ‘cause’). Check if the outcomes were measured in the same way. Same instrument or scale used? Same measurement timing? Same measurement procedures and instructions? | /2 |
| Were outcomes measured in a reliable way? | Unreliability of outcome measurements is one threat that weakens the validity of inferences about the statistical relationship between the ‘cause’ and the ‘effect’ estimated in a study exploring causal effects. Unreliability of outcome measurements is one of different plausible explanations for errors of statistical inference with regards to the existence and the magnitude of the effect determined by the treatment  (‘cause’). Check the details about the reliability of measurement such as the number of raters, training of raters, the intra-rater reliability, and the inter-raters reliability within the study (not to external sources). This question is about the reliability of the measurement performed in the study, it is not about the validity of the measurement instruments/scales used in the study. [Note: Two other important threats that weaken the validity of inferences about the statistical relationship between the ‘cause’ and the ‘effect’ are low statistical power and the violation of the assumptions of statistical tests. These other threats are not explored within Question 8, these are explored within Question 9. | /2 |
| Was appropriate statistical analysis used? | Inappropriate statistical analysis may cause errors of statistical inference with regards to the existence and the magnitude of the effect determined by the treatment (‘cause’). Low statistical power and the violation of the assumptions of statistical tests are two important threats that weakens the validity of inferences about the statistical relationship between the ‘cause’ and the ‘effect’. Check the following aspects: if the assumptions of statistical tests were respected; if appropriate statistical power analysis was performed; if appropriate effect sizes were used; if appropriate statistical procedures or methods were used given the number and type of dependent and independent variables, the number of study groups, the nature of the relationship between the groups (independent or dependent groups), and the objectives of statistical analysis (association between variables; prediction; survival analysis etc.). | /2 |
|  | Total score | /18 |

**Table 5.** Joanna Briggs Institute critical appraisal checklist for randomised, controlled studies with descriptors and scoring.

| Criterion | Description | Score |
| --- | --- | --- |
| Was true randomization used for assignment of participants to treatment groups? | The differences between participants included in compared groups constitutes a threat to the internal validity of a study exploring causal relationships. If participants are not allocated to treatment and control groups by random assignment there is a risk that the allocation is influenced by the known characteristics of the participants and these differences between the groups may distort the comparability of the groups. A true random assignment of participants to the groups means that a procedure is used that allocates the participants to groups purely based on chance, not influenced by the known characteristics of the participants. Check the details about the randomization procedure used for allocation of the participants to study groups. Was a true chance (random) procedure used? For example, was a list of random numbers used? Was a computer-generated list of random numbers used? | /2 |
| Was allocation to groups concealed? | If those allocating participants to the compared groups are aware of which group is next in the allocation process, that is, treatment or control, there is a risk that they may deliberately and purposefully intervene in the allocation of patients by preferentially allocating patients to the treatment group or to the control group and therefore this may distort the implementation of allocation process indicated by the randomization and therefore the results of the study may be distorted. Concealment of allocation (allocation concealment) refers to procedures that prevent those allocating patients from knowing before allocation which treatment or control is next in the allocation process. Check the details about the procedure used for allocation concealment. Was an appropriate allocation concealment procedure used? For example, was central randomization used? Were sequentially numbered, opaque and sealed envelopes used? Were coded drug packs used? | /2 |
| Were treatment groups similar at the baseline? | The differences between participants included in compared groups constitute a threat to the internal validity of a study exploring causal relationships. If there are differences between participants included in compared groups there is a risk of selection bias. If there are differences between participants included in the compared groups maybe the ‘effect’ cannot be attributed to the potential ‘cause’ (the examined intervention or treatment), as maybe it is plausible that the ‘effect’ may be explained by the differences between participants, that is, by selection bias. Check the characteristics reported for participants. Are the participants from the compared groups similar with regards to the characteristics that may explain the effect even in the absence of the ‘cause’, for example, age, severity of the disease, stage of the disease, co-existing conditions and so on? Check the proportions of participants with specific relevant characteristics in the compared groups. Check the means of relevant measurements in the compared groups (pain scores; anxiety scores; etc.). *[Note: Do NOT only consider the P-value for the statistical testing of the differences between groups with regards to the baseline characteristics.]* | /2 |
| Were those delivering treatment blind to treatment assignment? | If those delivering treatment are aware of participants’ allocation to the treatment group or to the control group there is the risk that they may behave differently with the participants from the treatment group and the participants from the control group, or that they may treat them differently, compared to the situations when they are not aware of treatment allocation and this may influence the implementation of the compared treatments and the results of the study may be distorted. Blinding of those delivering treatment is used in order to minimize this risk. Blinding of those delivering treatment refers to procedures that prevent those delivering treatment from knowing which group they are treating, that is those delivering treatment are not aware if they are treating the group receiving the treatment of interest or if they are treating any other group receiving the control interventions. Check the details reported in the article about the blinding of those delivering treatment with regards to treatment assignment. Is there any information in the article about those delivering the treatment? Were those delivering the treatment unaware of the assignments of participants to the compared groups? | /2 |
| Were outcomes assessors blind to treatment assignment? | If those assessing the outcomes are aware of participants’ allocation to the treatment group or to the control group there is the risk that they may behave differently with the participants from the treatment group and the participants from the control group compared to the situations when they are not aware of treatment allocation and therefore there is the risk that the measurement of the outcomes may be distorted and the results of the study may be distorted. Blinding of outcomes assessors is used in order to minimize this risk. Check the details reported in the article about the blinding of outcomes assessors with regards to treatment assignment. Is there any information in the article about outcomes assessors? Were those assessing the treatment’s effects on outcomes unaware of the assignments of participants to the compared groups? | /2 |
| Were treatment groups treated identically other than the intervention of interest? | In order to attribute the ‘effect’ to the ‘cause’ (the treatment or intervention of interest), assuming that there is no selection bias, there should be no other difference between the groups in terms of treatment or care received, other than the manipulated ‘cause’ (the treatment or intervention controlled by the researchers). If there are other exposures or treatments occurring at the same time with the ‘cause’ (the treatment or intervention of interest), other than the ‘cause’, then potentially the ‘effect’ cannot be attributed to the examined ‘cause’ (the investigated treatment), as it is plausible that the ‘effect’ may be explained by other exposures or treatments occurring at the same time with the ‘cause’ (the treatment of interest). Check the reported exposures or interventions received by the compared groups. Are there other exposures or treatments occurring at the same time with the ‘cause’? Is it plausible that the ‘effect’ may be explained by other exposures or treatments occurring at the same time with the ‘cause’? Is it clear that there is no other difference between the groups in terms of treatment or care received, other than the treatment or intervention of interest? | /2 |
| Was follow up complete and if not, were differences between groups in terms of their follow up adequately described and analyzed? | For this question, follow up refers to the time period from the moment of random allocation (random assignment or randomization) to compared groups to the end time of the trial. This critical appraisal question asks if there is complete knowledge (measurements, observations etc.) for the entire duration of the trial as previously defined (that is, from the moment of random allocation to the end time of the trial), for all randomly allocated participants. If there is incomplete follow up, that is incomplete knowledge about all randomly allocated participants, this is known in the methodological literature as the post-assignment attrition. As RCTs are not perfect, there is almost always post-assignment attrition, and the focus of this question is on the appropriate exploration of post-assignment attrition (description of loss to follow up, description of the reasons for loss to follow up, the estimation of the impact of loss to follow up on the effects etc.). If there are differences with regards to the loss to follow up between the compared groups in an RCT, these differences represent a threat to the internal validity of a randomized experimental study exploring causal effects, as these differences may provide a plausible alternative explanation for the observed ‘effect’ even in the absence of the ‘cause’ (the treatment or intervention of interest). When appraising an RCT, check if there were differences with regards to the loss to follow up between the compared groups.  If follow up was incomplete (that is, there is incomplete information on all participants), examine the reported details about the strategies used in order to address incomplete follow up, such as descriptions of loss to follow up (absolute numbers; proportions; reasons for loss to follow up) and impact analyses (the analyses of the impact of loss to follow up on results). Was there a description of the incomplete follow up (number of participants and the specific reasons for loss to follow up)? It is important to note that with regards to loss to follow up, it is not enough to know the number of participants and the proportions of participants with incomplete data; the reasons for loss to follow up are essential in the analysis of risk of bias; even if the numbers and proportions of participants with incomplete data are similar or identical in compared groups, if the patterns of reasons for loss to follow up are different (for example, side effects caused by the intervention of interest, lost contact etc.), these may impose a risk of bias if not appropriately explored and considered in the analysis. If there are differences between groups with regards to the loss to follow up (numbers/proportions and reasons), was there an analysis of patterns of loss to follow up? If there are differences between the groups with regards to the loss to follow up, was there an analysis of the impact of the loss to follow up on the results?  [Note: Question 8 is NOT about intention-to-treat (ITT) analysis; question 9 is about ITT analysis.] | /2 |
| Were participants analyzed in the groups to which they were randomized? | This question is about the intention-to-treat (ITT) analysis. There are different statistical analysis strategies available for the analysis of data from randomized controlled trials, such as intention-to-treat analysis (known also as intent to treat; abbreviated, ITT), per-protocol analysis, and as-treated analysis. In the ITT analysis the participants are analyzed in the groups to which they were randomized, regardless of whether they actually participated or not in those groups for the entire duration of the trial, received the experimental intervention or control intervention as planned or whether they were compliant or not with the planned experimental intervention or control intervention. The ITT analysis compares the outcomes for participants from the initial groups created by the initial random allocation of participants to those groups. Check if ITT was reported; check the details of the ITT. Were participants analyzed in the groups to which they were initially randomized, regardless of whether they actually participated in those groups, and regardless of whether they actually received the planned interventions? [Note: The ITT analysis is a type of statistical analysis recommended in the Consolidated Standards of Reporting Trials (CONSORT) statement on best practices in trials reporting, and it is considered a marker of good methodological quality of the analysis of results of a randomized trial. The ITT is estimating the effect of offering the intervention, that is, the effect of instructing the participants to use or take the intervention; the ITT it is not estimating the effect of actually receiving the intervention of interest.] | /2 |
| Were outcomes measured in the same way for treatment groups? | If the outcome (the ‘effect’) is not measured in the same way in the compared groups there is a threat to the internal validity of a study exploring a causal relationship as the differences in outcome measurements may be confused with an effect of the treatment (the ‘cause’). Check if the outcomes were measured in the same way. Same instrument or scale used? Same measurement timing? Same measurement procedures and instructions? | /2 |
| Was appropriate statistical analysis used? | Inappropriate statistical analysis may cause errors of statistical inference with regards to the existence and the magnitude of the effect determined by the treatment (‘cause’). Low statistical power and the violation of the assumptions of statistical tests are two important threats that weaken the validity of inferences about the statistical relationship between the ‘cause’ and the ‘effect’. Check the following aspects: if the assumptions of statistical tests were respected; if appropriate statistical power analysis was performed; if appropriate effect sizes were used; if appropriate statistical procedures or methods were used given the number and type of dependent and independent variables, the number of study groups, the nature of the relationship between the groups (independent or dependent groups), and the objectives of statistical analysis (association between variables; prediction; survival analysis etc.). | /2 |
| Was the trial design appropriate for the topic, and any deviations from the standard RCT design accounted for in the conduct and analysis? | Certain RCT designs, such as the crossover RCT, should only be conducted when appropriate. Alternative designs may also present additional risks of bias if not accounted for in the design and analysis.  Crossover trials should only be conducted in people with a chronic, stable condition, where the intervention produces a short term effect (i.e. relief in symptoms). Crossover trials should ensure there is an appropriate period of washout between treatments.  Cluster RCTs randomize groups of individuals, forming ‘clusters.’ When we are assessing outcomes on an individual level in cluster trials, there are unit-of-analysis issues, as individuals within a cluster are correlated. This should be taken into account by the study authors when conducting analysis, and ideally authors will report the intra-cluster correlation coefficient.  Stepped-wedge RCTs may be appropriate when it is expected the intervention will do more good than harm, or due to logistical, practical or financial considerations in the roll out of a new treatment/intervention. Data analysis in these trials should be conducted appropriately, taking into account the effects of time. | /2 |
|  | Total score | /26 |
